# Targeting alveolar epithelial cell metabolism in pulmonary fibrosis: Pioneering an emerging therapeutic strategy

**DOI:** 10.3389/fcell.2025.1608750

**Published:** 2025-06-25

**Authors:** Tengkun Dai, Yidan Liang, Xin Li, Jiamin Zhao, Guangqin Li, Qihong Li, Lin Xu, Juanjuan Zhao

**Affiliations:** ^1^ Department of Immunology, Zunyi Medical University, Zunyi, Guizhou, China; ^2^ Key Laboratory of Gene Detection and Treatment of Guizhou province, Zunyi, Guizhou, China

**Keywords:** pulmonary fibrosis, metabolic reprogramming, alveolar epithelial cells, energy metabolism, pathogenesis, glucose metabolism, lipid metabolism, amino acid metabolism

## Abstract

Pulmonary fibrosis (PF) is a chronic and progressive lung disease, characterized by excessive deposition of fibrotic connective tissue within the lungs. Advances in transcriptomics, proteomics, and metabolomics have enhanced our understanding of PF’s pathogenesis. Recent studies have indicates that metabolic abnormalities in alveolar epithelial cells (AECs) play a central role in the pathogenesis of PF. Metabolic reprogramming of AECs affects cellular senescence, endoplasmic reticulum stress, and oxidative stress in AECs, while also promoting fibrotic progression through various signaling pathways. This review focuses on therapeutic strategies targeting the metabolism of AECs. It comprehensively explores the role of metabolic pathways through glucose metabolism, lipid metabolism, and amino acid metabolism in the pathogenesis of PF, aiming to provide novel theoretical support and research perspectives for preventing and treating pulmonary fibrosis.

## Graphical Abstract

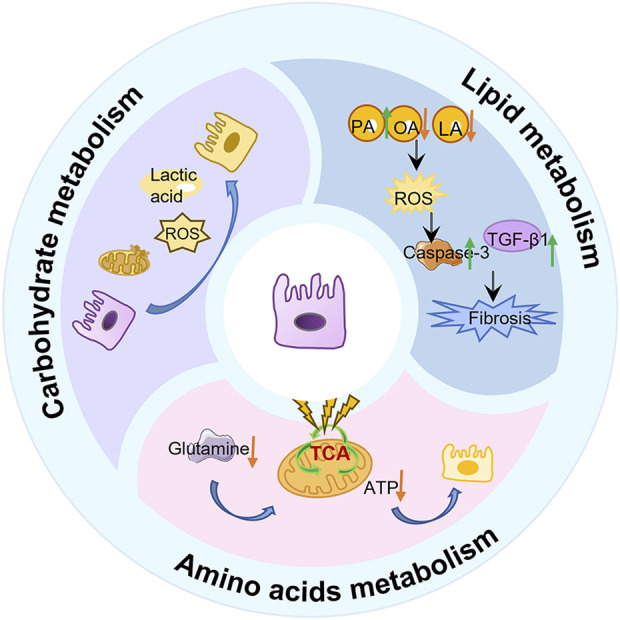

## 1 Introduction

Pulmonary fibrosis (PF) is a chronic, progressive, and irreversible lung disease (Koudstaal et al., 2023), encompassing idiopathic pulmonary fibrosis (IPF), connective tissue disease-related interstitial lung disease (ILD), and many other types (Smith, 2016). The disease is characterized by diffuse progressive remodeling of the lung parenchyma with extracellular matrix deposition and irreversible scar formation, which severely impairs the respiratory function of patients and even threatens their lives (Koudstaal et al., 2023). Nidanib and pirfenidone, both approved by the FDA in 2014, are the primary antifibrotic therapeutic agents currently used to treat IPF. However, although these drugs can slow the decline in lung function in IPF patients, they do not improve lung function or achieve a complete cure (Salisbury and Wijsenbeek, 2021). Therefore, it is particularly important to explore new therapeutic strategies. Recent research into the mechanisms underlying pulmonary fibrosis has identified alveolar epithelial cells (AECs) as key drivers in the disease’s development (Katzen and Beers, 2020).

**FIGURE 1:**
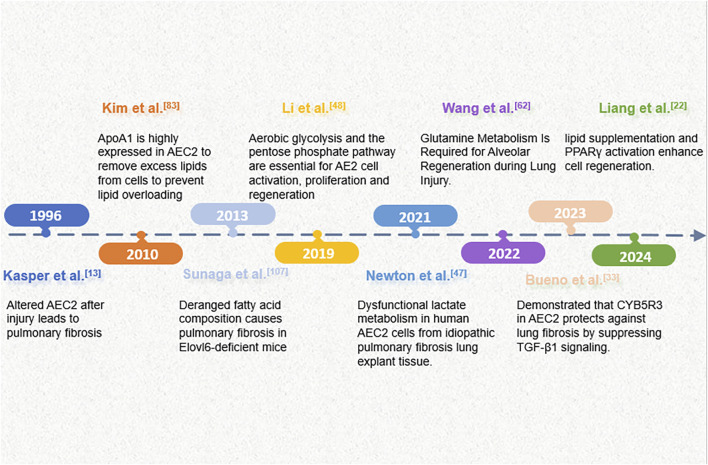
An overview of key events in the progress of research on metabolic alterations in AECs in pulmonary fibrosis.

AECs consist of 2 cell types, alveolar epithelial type I cells (AEC1), and alveolar epithelial type II cells (AEC2) (Motohashi and Yamamoto, 2004; Katzen and Beers, 2020; Wong and Johnson, 2013). These cells are critical for maintaining the lung structure and function. AEC1 cells cover approximately 95% of the alveolar surface and are responsible for gas exchange and alveolar fluid regulation (Maina and West, 2005; Johnson et al., 2002). Although AEC2 cells occupy only about 5% of the alveolar surface area, they are highly specialized and metabolically active, containing a dense array of subcellular organelles. Importantly, AEC2 cells serve as the major progenitor cells in the alveoli, capable of differentiating into AEC1 cells for alveolar repair and proliferating to support self-renewal (Fehrenbach, 2001). Notably, dysfunction of AEC2 cells is closely related to the pathogenesis of ILD and IPF (Parimon et al., 2020). However, the mechanisms by which AEC2 cell dysfunction contributes to these diseases remain a challenging question (Figure 1).

During the progression of PF, the morphology and function of AECs undergo significant alterations involving complex processes such as abnormal cell metabolism, apoptosis, and necrosis (Roque and Romero, 2021; Kasper and Haroske, 1996). In particular, metabolic abnormalities are critical biological processes in PF. Metabolic abnormalities refer to the disturbances of intracellular substance metabolism, potentially hindering cellular energy supply and the synthesis of vital molecules (Geng et al., 2022; Huo et al., 2024). During the PF process, metabolic pathways such as glucose, fatty acids, and amino acids are reprogrammed in AECs, thereby affecting cellular energy production, growth, and survival (Roque and Romero, 2021).

To gain a deeper understanding of the pathogenesis of pulmonary fibrosis and to provide theoretical support for future therapeutic strategies, increasing attention is being directed toward the role of metabolic abnormalities in AECs. This review will provides a comprehensive overview of the role of AECs, especially AEC2 metabolic abnormalities, in the pathogenesis of pulmonary fibrosis. We expect that this review will provide valuable references for future studies and new ideas and approaches for the treatment of pulmonary fibrosis.

## 2 Studies on the role of metabolic abnormalities in alveolar epithelial cells during pulmonary fibrosis

The lung is a metabolically active organ that plays an important role, though its importance is often underestimated. With the development of transcriptomics, proteomics, and metabolomics, metabolic reprogramming has been gradually recognized as one of the central drivers of PF pathology. Recent studies have shown that the development of PF is closely associated with altered metabolic pathways in AECs, especially dysregulation of glucose metabolism, lipid metabolism, and amino acid metabolism, which accelerates the pathologic process.

### 2.1 Lipid metabolism and pulmonary fibrosis

Due to the abundance of lipids in lung tissue, lipid metabolism and its regulation are critical for lung physiological function (Burgy et al., 2022; Rajesh et al., 2023). In the lungs, lipids are not only a major component of cell membranes but also involved in physiological processes such as energy storage, signaling, and inflammatory responses (Wasnick et al., 2023; Berry et al., 2017). PF is characterized by a unique circulating metabolic profile with elevated levels of nonesterified fatty acids, long-chain acylcarnitines, and ceramides, indicating a more catabolic environment for lipid mobilization and metabolism (Summer et al., 2024).

#### 2.1.1 Lipid metabolism in pulmonary homeostasis and fibrosis

AEC2s are the lung’s chief surfactant-producing, lipid-metabolizing cells (Wasnick et al., 2023). They secrete a lipoprotein surfactant (∼90% lipid, rich in phosphatidylcholine and cholesterol) that lowers alveolar surface tension and maintains barrier integrity (Rindlisbacher et al., 2018; Liang et al., 2024; Chung et al., 2019; Whitsett et al., 2015; Ikegami et al., 1985).In pulmonary fibrosis, however, AEC2 lipid homeostasis collapses: single-cell profiles of IPF lungs show broad downregulation of genes for fatty-acid synthesis, β-oxidation and cholesterol biosynthesis in AEC2s, yielding lipid-poor cells and surfactant insufficiency (Rindlisbacher et al., 2018; Liang et al., 2024; Chung et al., 2019).In recent years, studies have shown that the abnormal lipid characteristics present in the serum of IPF patients and mouse IPF models are mainly the release of damaged AEC2, which subsequently participates in the fibrotic process (Yang et al., 2024). Among them, the metabolism of glycerophospholipids and choline underwent significant changes. The study by Baker DL et al. (Baker et al., 2006) identified lysophosphatidic acid, (LysoPA) in the serum of patients with IPF. LysoPC is the precursor of LPA, which is a bioactive glycerophospholipid. LPA induces pulmonary, renal and liver fibrosis through epithelial cell death, vascular leakage and fibroblast migration and proliferation (Alsafadi et al., 2017; Sakai et al., 2017; Kaffe et al., 2017; Tager et al., 2008). It is worth noting that after lung injury, dipalmitoyl phosphatidylcholine (DPPC), as the main surfactant lipid component, is degraded into LysoPC through the phospholipase A2 activity of AEC2, further exacerbating the process of pulmonary fibrosis (Beers et al., 2017; Shi et al., 2024). This change in lipid secretion profile is closely related to the decline of lung function, among which the depletion of phosphatidylcholine (PC) is particularly prominent. The level of PC in bronchoalveolar lavage fluid is significantly decreased, and it shows a significant negative correlation with the decline of lung compliance. Meanwhile, the research by Shi X et al. found that AEC2 can take up cholesterol from extracellular low-density lipoprotein through the low-density lipoprotein receptor (LDLR) (Shi et al., 2022). Adipocytes can transport lipids to AEC2 cells through the parathyroid hormone-associated protein (PTHRP) signaling pathway, which is activated by tensile-sensitive AEC2 cells and guides the differentiation of mesenchymal and alveolar epithelial cells (Chao et al., 2015; Torday and Rehan, 2002). These lipids may contribute to the synthesis of AEC2 surfactant lipids. To sum up, all these results indicate that the surfactant phospholipids in PF decrease, thereby exacerbating the course of IPF.

**FIGURE 2:**
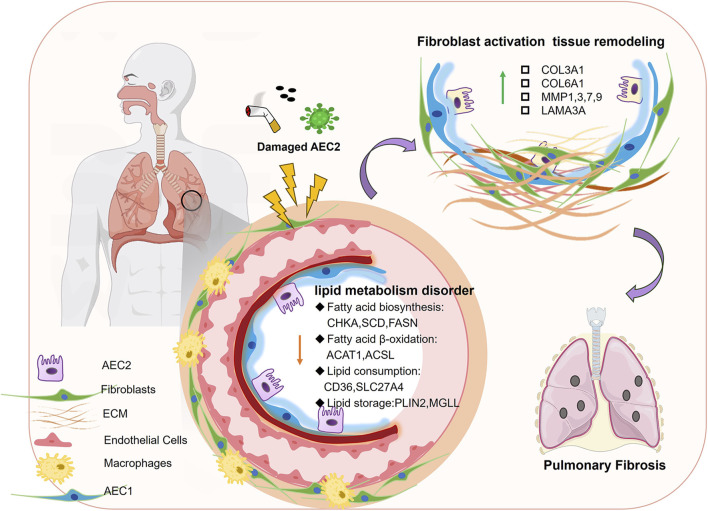
Disturbed lipid metabolism in AECs in PF. When AEC2 is damaged by smoke, viruses, etc., fatty acid biosynthesis, fatty acid β-oxidation, and other lipid-related components are downregulated, leading to fibroblast activation tissue remodeling, which in turn leads to pulmonary fibrosis.

Furthermore, with the development of transcriptome and single-cell sequencing, the research results on the role of AEC2 lipid metabolic homeostasis in the development of IPF have been reported at both the cellular and organ levels. For example, Rindlisbacher et al. (2018) identified 500 downregulated genes involved in lipid metabolism, cholesterol handling, and steroid metabolism in AEC2 cells from PF patients by metabolomics analysis. Similarly, Liang et al. (2024) observed dysregulated expression of lipid metabolism genes in AEC2 cells from homeostatic, aged, and young mice following lung injury, as well as in PF patients, using scRNA-seq analysis. In particular, genes related to fatty acid synthesis (e.g., CHKA, SCD, FASN, etc.) and fatty acid β-oxidation (β-oxidation, a mitochondrial process that breaks down fatty acids to generate energy; e.g., ACAT1, ACSL) were significantly downregulated in AEC2 cells from IPF patients (Chung et al., 2019) (Figure 2).

#### 2.1.2 Organelle dysfunction and lipid metabolic dysregulation in pulmonary fibrosis

Notably, the regulation of lipid homeostasis in AECs also closely linked to organelle integrity (Gunasekara et al., 2005; Mesmin, 2016). Studies have reported that endoplasmic reticulum (ER) stress is significantly increased in human and mouse AEC2 cells suffering from pulmonary fibrosis. Currently, a recognized adaptive response to ER stress is the induction of lipid synthesis by affected cells. For example, a negative correlation between the expression of lipid synthase and the expression of ER stress markers was observed in AEC2 cells from a mouse PF model (Romero et al., 2018a). Specifically, under PF conditions, increased ER stress in AEC2 resulted in impaired lipid synthesis, in particular a reduction in unsaturated fatty acid synthesis mediated by stearoyl coenzyme A desaturase 1 (SCD1) Reduced SCD1 activity not only impeded resolution of endoplasmic reticulum stress, but also exacerbated cellular dysfunction, thereby triggering a fibrotic response (Romero et al., 2018a).

In addition, mitochondrial dysregulation critically impacts lipid metabolism in PF. Mitochondrial dynamics—a process regulated by fission and fusion events—govern cellular energy homeostasis and functional adaptation (Toyama et al., 2016; Chen et al., 2003). Key mediators include the outer membrane GTPases MFN1 and MFN2, which coordinate mitochondrial fusion and directly regulate phospholipid/cholesterol synthesis in AEC2 (Shi et al., 2022; Chen et al., 2003). Disruption of this balance impairs surfactant lipid production, compromising epithelial barrier integrity and accelerating PF progression (Shi et al., 2022). Concurrently, diminished expression of CYB5R3 (a redox enzyme modulating NAD^+^/NADH equilibrium) in PF-associated AEC2 exacerbates mitochondrial dysfunction and aberrantly activates TGF-β1 signaling. This dual role positions CYB5R3 upregulation as a potential therapeutic strategy to restore epithelial stem cell function and mitigate metabolic derangements in PF (Rahaman et al., 2017; Hall et al., 2011; Bueno et al., 2023).

**FIGURE 3:**
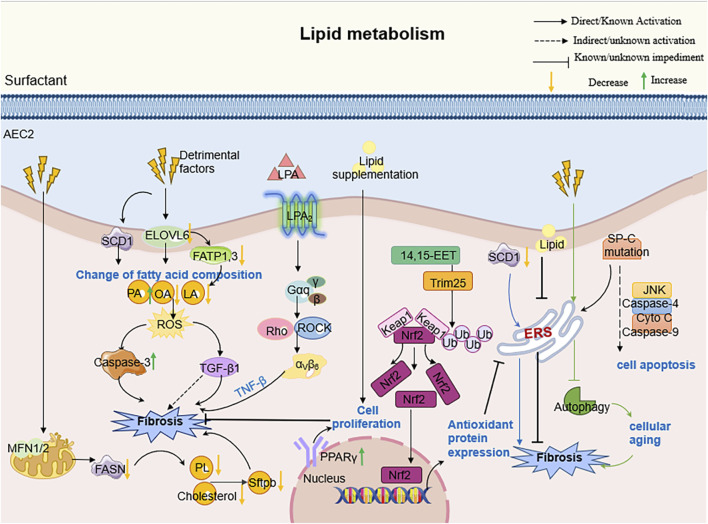
The molecular mechanism of AEC2 lipid metabolism in PF. Specifically speaking, Elovl6 deficiency led to changes in the composition of fatty acid content in AEC2 cells, including an increase in C16 PA and a decrease in C18 OA and C18 LA, which triggered a disturbance in lipid metabolism; this metabolic dysregulation further contributed to the progression of pulmonary fibrosis by inducing apoptosis, ROS production, TGF-β1 and other pathways.

Recently, studies have found that autophagy-mediated metabolic reprogramming counteracts the fibrotic development process by regulating lipid fluxes during injury (Rahaman et al., 2017; Hall et al., 2011; Bueno et al., 2023; Galluzzi et al., 2017; Levine and Kroemer, 2019). In lung injury models, active autophagy in AEC2s shifts metabolism away from lipid storage toward energy production: autophagy downregulates fatty acid and triglyceride biosynthesis while upregulating glycolysis and β-oxidation to meet bioenergetic needs for repair (Araya et al., 2013). This “lipophagic” reprogramming helps clear toxic lipid intermediates and provides substrates for new membrane synthesis, thereby promoting AEC2 proliferation, surfactant regeneration, and barrier repair (Araya et al., 2013; Margaritopoulos et al., 2013). In contrast, impaired autophagy leads to accumulation of lipid byproducts and oxidative stress, which impairs regeneration and amplifies TGF-β1 signaling (Margaritopoulos et al., 2013; Li X. et al., 2020; Miller and Freeze, 2003; Hamanaka and Mutlu, 2021). Thus, ER stress, mitochondrial injury, and autophagy converge on AEC2 lipid metabolism: balanced regulation of lipid synthesis, desaturation, and degradation is essential for surfactant homeostasis and for restraining TGF-β1-driven fibrotic remodeling. (Figure 3).

### 2.2 Carbohydrate metabolism and pulmonary fibrosis

Carbohydrate metabolism is critically involved in PF, influencing cellular energy production, redox balance, and alveolar epithelial function. Dysregulated glycolysis, mitochondrial dysfunction, and impaired autophagy contribute to metabolic imbal-ances that drive fibrotic progression.

#### 2.2.1 Dysregulated glycolysis and lactate accumulation in pulmonary fibrosis

Carbohydrate metabolism, a cornerstone of cellular energy supply and homeostasis, is profoundly altered in PF. Under physiological conditions, lung tissues exhibit a unique metabolic preference: approximately 40% of glucose is converted to lactate via glycolysis despite adequate oxygen availability—a phenomenon reminiscent of the “Warburg effect” observed in cancer (Korfei et al., 2008; Lee et al., 2004; Hui et al., 2017; Bassett and Fisher, 1976). In PF, this metabolic signature becomes pathological, with lactate levels in fibrotic lungs tripling compared to healthy tissues, driven by both overproduction and impaired clearance (Kottmann et al., 2012; Burman et al., 2018). AEC2 emerge as central players in this dysregulation. While healthy AEC2 utilize lactate for mitochondrial ATP synthesis (Lee et al., 2004), their PF counterparts undergo metabolic reprogramming characterized by defective oxidative phosphorylation and a shift toward glycolytic dominance. This shift is mediated by isoform-specific upregulation of lactate dehydrogenase (LDH): PF-associated AEC2 predominantly express LDH4/LDH5 isoforms that favor pyruvate-to-lactate conversion, contrasting with the LDH2/LDH3 dominance in non-fibrotic cells (Kottmann et al., 2012). The resultant lactate accumulation exerts multifaceted pro-fibrotic effects. It enhances TGF-β-induced myofibroblast differentiation through pH-dependent mechanisms (Newton et al., 2021), disrupts NAD^+^/NADH redox balance to accelerate cellular senescence (Li et al., 2019; Valdivieso et al., 2019; Massip-Copiz et al., 2021; Cui et al., 2022), and activates ER stress via the ATF4-Chop axis, triggering AEC2 apoptosis (Sun et al., 2024). Notably, therapeutic interventions targeting LDHA—a key glycolytic enzyme—reverse lactate-driven acidification and restore oxidative metabolism in PF models, mirroring findings in cystic fibrosis where CFTR mutations similarly elevate lactate through mitochondrial dysfunction (Newton et al., 2021; Valdivieso et al., 2019; Massip-Copiz et al., 2021). Collectively, these insights reposition lactate not merely as a metabolic byproduct, but as a pivotal mediator bridging glycolytic dysregulation to fibrotic tissue remodeling.

#### 2.2.2 Carbohydrate metabolism disorder and imbalance of energy homeostasis in pulmonary fibrosis

Energy metabolism dysregulation is a hallmark of PF, critically influencing disease progression. AEC2 responsible for surfactant production, exhibit profound mitochondrial dysfunction in PF, characterized by structural abnormalities (e.g., swelling), impaired mitophagy, and diminished biogenesis (Yan et al., 2023). These defects disrupt oxidative phosphorylation, reducing ATP synthesis while amplifying mitochondrial ROS (mtROS) generation—a toxic byproduct that perpetuates mitochondrial DNA damage and lipid peroxidation, thereby exacerbating both energetic crisis and fibrotic remodeling (Larson-Casey et al., 2020; Kim et al., 2016).

**FIGURE 4:**
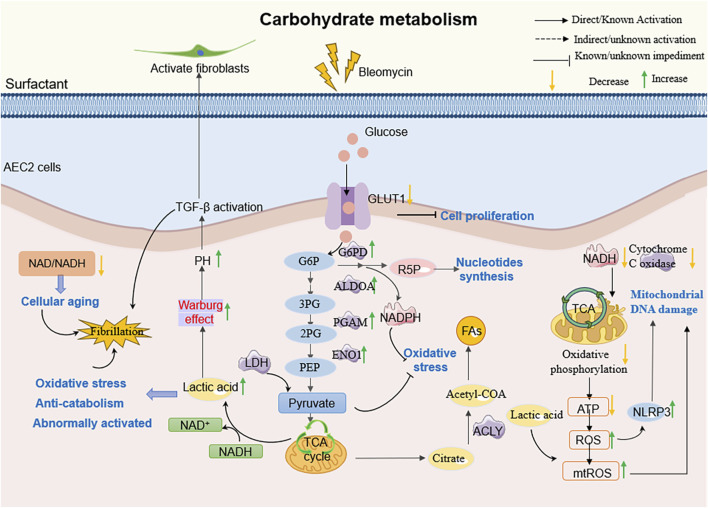
The molecular mechanism of AEC2 carbohydrate metabolism in PF. For example, AEC2 cells in patients with PF, on the other hand, tend to undergo inefficient oxidative metabolism, produce more glycolytic lactic acid, and increase TGF-β expression, thereby promoting fibrogenesis.

Central to mitochondrial quality control is the PINK1/Parkin-mediated mitophagy pathway. In PF, attenuated PTEN expression in lung epithelia sustains AKT activation and TGF-β signaling, further compromising epithelial integrity (Bueno et al., 2015). Concurrently, IL-17A exacerbates PF susceptibility by suppressing PINK1/Parkin activity, which disrupts mitophagy and amplifies apoptosis through dysregulated TGF-β, STAT3, and NF-κB pathways (Bueno et al., 2015). Beyond apoptosis regulation, autophagy sustains AEC2 proliferative capacity post-injury. Enhanced autophagy upregulates glycolytic enzymes (e.g., PGAM, ENO1, ALDOA) and glucose-6-phosphate dehydrogenase (G6PDX), boosting NADPH production to counteract oxidative stress while fueling alveolar repair (Li X. et al., 2020). This metabolic adaptation ensures redox homeostasis and maintains AEC2 regenerative potential. (Figure 4).

Glucose metabolism further modulates PF progression through key regulators. GLUT1 deficiency during lung injury impairs AEC2 proliferation despite compensatory upregulation of glycolysis and the pentose phosphate pathway (Newton et al., 2021). Similarly, diminished SIRT3 activity-a mitochondrial deacetylase vital for mtDNA integrity-promotes mitochondrial dysfunction and AEC apoptosis, highlighting the interplay between acetylation states and metabolic resilience (Xiao et al., 2022; Li J. et al., 2020; Bueno et al., 2018a). Collectively, these findings underscore that restoring AEC2 energy metabolism homeostasis represents a pivotal therapeutic avenue to mitigate fibrotic progression.

### 2.3 Amino acid metabolism and pulmonary fibrosis

Amino acid metabolism is a crucial biochemical process in living organisms, encompassing various aspects of synthesis, degradation, and regulation. This process is tightly controlled and closely linked to physiological functions such as cell growth, immune responses, and neurotransmission.

#### 2.3.1 Glutamine metabolism and AEC2 dysfunction

**FIGURE 5:**
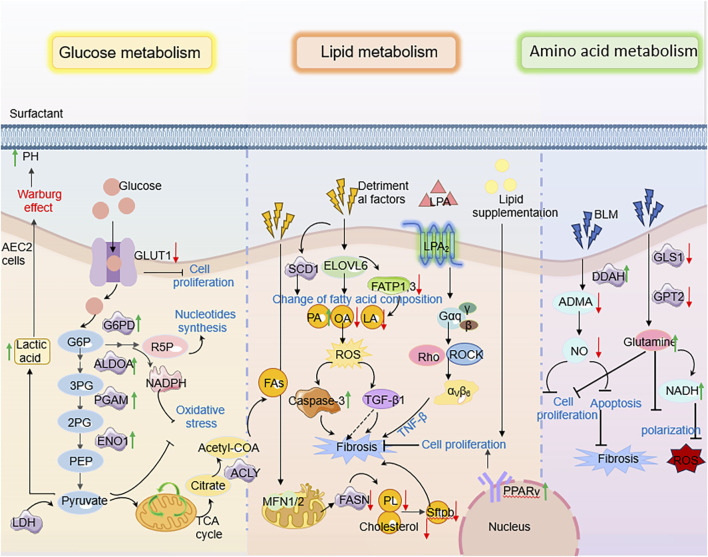
The molecular mechanism of AEC2 metabolism in PF.

Glutamine plays a pivotal role in antioxidant defense through multiple mechanisms, including the production of NADPH, which regulates the synthesis of ROS detoxification enzymes, and serves as a core component of the cellular antioxidant glutathione. Studies have demonstrated that significant metabolic changes occur during glucose metabolism, necessitating cells to rely on alternative metabolic fuels like glutamine to support mitochondrial respiration and metabolite production, thereby inhibiting the progression of PF. Glutamine, an essential metabolic substrate, exhibits significantly elevated requirements in various pathological states (Vigeland et al., 2019; Wise and Thompson, 2010). Notably, the expression of key enzymes involved in glutamine metabolism (e.g., GLS1, GOT2, OGDH, SUCLG) is downregulated in AEC2 from PF patients and in bleomycin-induced mouse models of pulmonary fibrosis. This downregulation leads to inhibited glutamine metabolism and, consequently, impairs the proliferation and differentiation of AEC2 cells (Wang et al., 2022). Additionally, Shaghaghi et al. (Shaghaghi et al., 2021) found that glutamine supplementation reduces the cytotoxicity of bleomycin on AEC2 cells by restoring mitochondrial respiration in alveolar epithelial cells. Further investigations revealed that glutamine addition increases intracellular metabolite levels, including various tricarboxylic acid (TCA) cycle intermediates and the glycolytic intermediate lactate, and is associated with reduced DNA damage and cell death induced by bleomycin. These findings provide new insights into the role of amino acid metabolism in pulmonary fibrosis (Pullamsetti et al., 2011) (Figure 5).

#### 2.3.2 Arginine metabolism and nitric oxide signaling

Abnormalities in amino acid metabolism are particularly critical in the context of PF, especially the pathways involved in nitric oxide (NO) production. Asymmetric dimethylarginine (ADMA) is a by-product of arginine metabolism and acts as an inhibitor of endogenous NO synthase, which regulates the production of NO. Clinical data analysis shows that patients with IPF report higher levels of alveolar nitric oxide, which are closely related to the severity of the disease (Zhao et al., 2017; Masri, 2010). Pullamsetti et al. (2011) discovered that dimethylarginine dimethylaminohydrolase (DDAH) activity is increased in lung AEC2 cells of bleomycin-induced fibrotic mice and IPF patients due to an increase in TGF-β1 and IL-6. In AEC2 cells cultured from bleomycin-induced fibrotic mouse lung, inhibition of DDAH suppresses cell proliferation and induces apoptosis in an ADMA-dependent manner; it also reduces collagen production by fibroblasts in an ADMA-independent but transforming growth factor/SMAD-dependent manner (Pullamsetti et al., 2011; Masri, 2010).

#### 2.3.3 Amino acid biomarkers and diagnostic potential

Recent studies suggest that amino acid metabolism may offer a rapid and non-invasive way to better characterize IPF and simplify the diagnostic process. Gaugg et al. (2019) reported increased levels of proline, a key component of collagen, in the lung tissues of patients with IPF compared to healthy controls, which was attributed to an increased production of proline via the ornithine aminotransferase (OAT) pathway. Previous research has shown elevated lung levels of 4-hydroxyproline, as well as polyamine putrescine and spermidine, in IPF patients, supporting the notion that ornithine metabolism is dysregulated in PF (Zhao et al., 2017). In addition, Gaugg et al. (2019) further verified a significant increase in collagen-related amino acids (e.g., proline and 4-hydroxyproline) in IPF patients, suggesting that collagen metabolism and elevated extracellular matrix turnover are key factors in PF progression. By using real-time breath analysis technology (SESI-MS), this study successfully detected a variety of amino acids in the breath of PF patients, including proline, alanine, and lysine. This noninvasive assay is simpler and more clinically useful compared to traditional lung biopsies, showing potential as a diagnostic tool for PF. Notably, higher levels of certain amino acids, such as branched-chain amino acids (valine, leucine, and isoleucine), were associated with less severe disease damage, higher diffusion capacity of the lungs for carbon monoxide (DLco%), and lower composite physiological index (CPI). In addition to serving as key substrates for energy metabolism and protein synthesis, these amino acids regulate growth and energy metabolism by activating the mTOR pathway (Karna et al., 2020; Romero et al., 2016; Platé et al., 2020). mTOR is over-activated in fibroblasts and epithelial cells in the PF, making the relationship between their elevated circulating levels and mTOR activity in the lungs a subject worthy of further exploration.

In summary, the function of AEC2 cells is precisely regulated by multiple organelles and proteins to maintain normal amino acid metabolism. In the context of PF, disruption of these regulatory mechanisms may lead to abnormal metabolism, which in turn promotes the process of pulmonary fibrosis. Future studies need to further delve into the specific mechanisms of the role of these metabolic processes in pulmonary fibrosis, to provide new ideas and approaches for the treatment of the disease.

### 2.4 Effects of metabolic abnormalities on alveolar epithelial cells

Alveolar epithelial cells, particularly AEC2 cells, are critical for maintaining lung physiologic function and coping with injury (de la et al., 2020). Under normal physiological conditions, these cells ensure the smooth functioning of the respiratory system by precisely regulating gas exchange and secreting lung surfactant (Delbrel et al., 2018). However, metabolic abnormalities can severely impair their function.

Firstly, metabolic abnormalities may lead to the destabilization of membrane lipid components, which in turn affects cell membrane integrity and function. This instability may be related to the enhanced activity of cytochrome P450 reductase (POR), which promotes the process of lipid peroxidation by accelerating the cycling between Fe2^+^ and Fe3^+^ (Wu and Song, 2020). Meanwhile, POR and CYB5R1 can also transfer electrons from NAD(P)H to oxygen to generate hydrogen peroxide and generate reactive hydroxyl radicals via the Fenton reaction, which further exacerbates lipid peroxidation (LPO), thus disrupting the integrity of cell membranes (Zou et al., 2020). Moreover, metabolic disorders can impair mitochondrial function, leading to reduced energy production (Rangarajan et al., 2017; Álvarez et al., 2017). For example, Bueno et al. (2023) found that ATP production was reduced and mitochondrial ROS production was increased in the lung tissues of patients with PF. Metabolic disorders may also lead to a reduced ability of alveolar epithelial cells to respond to oxidative stress and other external insults. For example, Xie et al. (2015) discovered that low-density lipoprotein (LDL) enters lung cells through its receptor (LDLR) and LRP1 to release free cholesterol. Excess free cholesterol is stored in lipid droplets, triggering lung cell inflammation and fibrosis manifested by overexpression of collagen, TGF-β1, TNF-α, and MMPs. This reduced responsiveness may be associated with activation of the apoptotic pathway and increased inflammatory response, which affects the stability of the alveolar epithelial layer. In IPF, necrotic apoptosis predominantly occurs in AEC2 cells, and the release of cellular contents may further contribute to the aggregation of pro-inflammatory cells and amplified tissue damage (Mamazhakypov et al., 2019). Overall, the effects of metabolic abnormalities on alveolar epithelial cells are comprehensive and far-reaching, involving multiple dimensions of cellular structure, function, energy homeostasis, and stress response. An in-depth understanding of these effects not only helps to reveal the association between metabolism and lung diseases, but also provides new ideas for the treatment of related diseases.

## 3 Strategies and perspectives for the treatment of pulmonary fibrosis based on metabolic abnormalities

In recent years, new therapeutic strategies have emerged through the in-depth studies of the pathogenesis of pulmonary fibrosis. Increasing evidence suggests that an imbalance in metabolic homeostasis, caused by repetitive alveolar epithelial injury, is a potential mechanism underlying IPF. Below, we focus on therapeutic strategies addressing AEC2 metabolic abnormalities and analyze their potential value and challenges (Figure 5).

### 3.1 AEC2-based therapeutic strategies for lipid metabolism

Dysregulated lipid metabolism in AEC2 drives pulmonary fibrosis by impairing surfactant synthesis and amplifying oxidative stress. Mitochondrial dynamics imbalance (e.g., MFN1/MFN2 dysfunction) and defective lipid homeostasis exacerbate lipid peroxidation and epithelial barrier disruption. Emerging interventions aim to restore lipid metabolism balance through mitochondrial fusion enhancers, CYB5R3 redox modulation, and autophagy-driven lipid clearance, offering potential to suppress fibrotic signaling and promote alveolar repair.

#### 3.1.1 Lipid metabolism enzyme-mediated AEC2 dysfunction and anti-fibrotic targets

Lipid metabolism plays a crucial role in the function of AEC2 cells and the development of pulmonary fibrosis. Studies have shown that lipid metabolism not only affects energy metabolism and membrane stability in AEC2 cells but also is directly and closely related to the progression of the fibrotic process. Among them, acetyl coenzyme A synthase short-chain family member 3 (ACSS3) regulates ECM deposition by reducing fatty acid oxidation and enhancing anaerobic glycolysis through carnitine palmitoyltransferase type I alpha (CPT1A) deficiency. Therefore, ACSS3 is considered a potential therapeutic target for pulmonary fibrosis (Wang et al., 2024).

Lipid elongation and desaturation, mediated by enzymes like Elovl6 and stea-royl-coenzyme A desaturase (SCD), are critical for fatty acid biosynthesis. Elovl6 defi-ciency alters fatty acid composition in AEC2s, increasing palmitate (C16:0) and reduc-ing oleic acid (C18:1n-9) and linoleic acid (C18:2n-6), leading to metabolic disturb-ances that promote apoptosis, ROS production, and TGF-β1 signaling (Chen et al., 2023; Green et al., 2010; Moon et al., 2001; Matsuzaka et al., 2012). Inhibition of lipid biosynthesis through targeted deletion of fatty acid synthase (FASN) or SCD1 ex-acerbates mitochondrial dysfunction, ER stress, and fibrosis, while overexpression of FASN or activation of liver X receptor (LXR) agonists attenuates fibrotic progression (Chung et al., 2019; Shin et al., 2023). These findings underscore the importance of lipid metabolism in maintaining AEC2 function and limiting.

#### 3.1.2 Lipid metabolism-related proteins and therapeutic targets

Lipid synthesis is regulated by various factors, and beyond lipid synthases, lipid metabolism-related proteins play an important role in this process. Apolipoprotein A1 (APOA1), a major protein component of high-density lipoprotein (HDL), belongs to the serum apolipoproteins (Kim et al., 2010). Lee et al. (2013) found that APOA1, in AEC2 cells with PF, may inhibit the production of TGF-β1 and reduce the number of apoptotic cells by increasing the level of the lipid mediator LXA4, which may reduce early and established lung inflammation and fibrosis. Furthermore, Gordon et al. (2016) revealed that APOA1, in AEC cells in lung tissue, prevents lipid overload and removes excess lipids from the cells. This further emphasizes the important role of APOA1 in protecting against lung injury and fibrosis. With a deeper understanding of the role of lipid metabolism in PF, more studies are revealing the potential to intervene in the fibrotic process by modulating lipid metabolism. The finding that metformin, an AMPKα activator and lipid synthesis inhibitor, has been shown to be effective in reversing the process of established pulmonary fibrosis in mice further emphasizes the critical role of lipid metabolism in IPF (Zelcer and Tontonoz, 2006). In addition, recent studies have shown that the FDA-approved lipid-lowering drugs fenofibrate and ciprofibrate significantly attenuated the extent of pulmonary fibrosis and reduced collagen production in fibroblasts and myofibroblast differentiation in mice (Samah et al., 2012), providing new evidence for the use of lipid metabolism modifiers in the treatment of pulmonary fibrosis. At the same time, studies have begun to focus on the role of lipid receptors and lipid delivery systems in the treatment of pulmonary fibrosis. For example, preclinical studies have shown that blocking certain lipid receptors (e.g., GPR84, LPA1, or CysLT1), as well as activating GPR40, may have an anti pulmonary fibrosis effect. In addition, Gwinn et al. (2011) found that local delivery of liposomes consisting of L-α-phosphatidylcholine and cholesterol effectively alleviated bleomycin-induced lung injury in mice. Similarly, Kornilova et al. (2001) demonstrated that phosphatidylcholine liposomes promoted wound healing in a guinea pig surgical lung injury model. These studies further emphasize the important role of lipid metabolism regulation in the treatment of pulmonary fibrosis and provide new directions for future therapeutic strategies.

#### 3.1.3 Metabolic abnormalities, ER stress, and combined therapeutic strategies

Emerging evidence indicates that metabolic derangements and ER stress form a feed-forward loop that aggravates AEC2 dysfunction in pulmonary fibrosis. High-fat diets enriched in palmitic acid worsen bleomycin-induced fibrosis by driving lipid overload, AEC2 cell death, and unresolved ER stress, thereby potentiating TGF-β signaling and matrix deposition (Kornilova et al., 2001; Chu et al., 2019). Conversely, restoring balanced lipid metabolism in AEC2s can mitigate ER stress and fibrosis. For example, enhancement of SCD1 activity or supplementation with monounsaturated fatty acids not only replenishes surfactant phospholipids but also alleviates ER stress markers (BiP, CHOP) and reduces collagen accumulation in fibrotic lungs (Summer and Mora, 2019). Similarly, administration of epoxyeicosatrienoic acids (EETs) via the CYP2J2 pathway improves redox homeostasis in AEC2s, promotes Nrf2-dependent antioxidant responses, and indirectly stabilizes ER function by preserving phospholipid bilayer integrity (Zhang et al., 2023).

Therapeutically, combining lipid-centric strategies with ER stress modulators yields synergistic benefits. Citrus aurantium alkaline extract (CAE), for instance, activates ATF3/PINK1-mediated mitophagy, resets fatty-acid β-oxidation, and lowers ER stress, collectively enhancing AEC2 survival and reducing fibrotic remodeling. Likewise, overexpression of Sestrin2 in AEC2s suppresses ROS and pro-inflammatory cytokine release (TNF-α, IL-6, IL-1β), prevents lipid peroxidation, and curbs ER-stress–induced ferroptosis, thereby preserving surfactant synthesis and barrier integrity (Zhang et al., 2023; Wang et al., 2021). These findings underscore that targeted restoration of lipid metabolic homeostasis—in particular, promoting desaturation (via SCD1), supporting physiologic phospholipid pools, and enhancing lipophagy—can potently attenuate ER stress (Zhang et al., 2023; Wang et al., 2021; Dong et al., 2023) (Table 1).

**TABLE 1:** Overview of therapeutic targets and associated pathways targeting AEC2 in pulmonary fibrosis.

Metabolic type	Therapeutic targets	Relevant pathway	References
	LDH5	Maintenance of lactate metabolism homeostasis	Newton et al. (2021)
	MiR-200	Regulation of LDHA maintains lactate metabolism homeostasis	Moimas et al. (2019)
	NAD/NADH	Maintaining cellular energy metabolism and redox balance	Cui et al. (2022)
Carbohydrates metabolism	PINK1	Regulates mitochondrial homeostasis and reduces accumulation of damaged mitochondria	Bueno et al. (2015)
	ATF3	Restoration of PINK1 expression levels and maintenance of mitochondrial homeostasis by inhibiting its expression	Bueno et al. (2018a)
	IL-17A	Increasing PINK1 levels and maintaining mitochondrial homeostasis by inhibiting its expression	Xiao et al. (2022)
	EETs	Inhibition of ER stress, thereby mitigating senescence in AECs	Zhang et al. (2023)
	APOA1	Increased levels of the lipid mediator LXA4 inhibit TGF-β1 production and reduce apoptosis	Lee et al. (2013)
lipid metabolism	ACSS3	Its overexpression inhibited the excessive deposition of ECM	Wang et al. (2024)
	Elovl6	Promotes apoptosis and reactive oxygen species and impairs cellular uptake of long-chain FAs	Levine and Kroemer (2019)
	FASN	Deficiency inhibits lipid biosynthesis, leading to AEC2 mitochondrial dysfunction, epithelial ER stress	Chung et al. (2019)
	PPARγ	Promotes AEC2 progenitor cell renewal	Liang et al. (2024)
	Mitofusin 1/2	Maintaining the stability of surfactant protein genes	Chung et al. (2019)
	Glutamine	Restoration of mitochondrial respiration in alveolar epithelial cells to reduce the cytotoxicity of bleomycin on AEC2	Shaghaghi et al. (2021)
Amino acids metabolism	DDAH	Reduced collagen production by fibroblasts	Pullamsetti et al. (2011)
	ADMA	Regulation of NO production	Pullamsetti et al. (2011)

### 3.2 AEC2-based therapeutic strategies for carbohydrate metabolism

Targeting AEC2 carbohydrate metabolism offers novel therapeutic avenues for pulmonary fibrosis. Dysregulated glycolysis and lactate overproduction in AEC2 exacerbate fibrosis by disrupting redox balance and impairing alveolar repair. Current strategies focus on inhibiting glycolytic enzymes (e.g., LDHA), enhancing mitochondrial oxidative phosphorylation, and modulating glucose transporters (e.g., GLUT1) to restore metabolic homeostasis and halt fibrotic progression.

#### 3.2.1 Lactate accumulation and targeting LDHA in pulmonary fibrosis

Emerging studies indicate that metabolic reprogramming in AEC2 contributes to lactate accumulation in pulmonary fibrosis, which exacerbates fibrotic progression by activating TGF-β/mTOR signaling and suppressing anti-fibrotic miRNAs such as the miR200 family (Acharya et al., 2019; Abedini et al., 2011; Gilbert et al., 2015; Song et al., 2022; Middleton et al., 2018; Zhang et al., 2016; Di Gregorio et al., 2020). LDHA, a key glycolytic enzyme upregulated in IPF-AEC2, has been identified as a critical driver of this process. Preclinical evidence demonstrates that LDHA inhibition via RNAi restores oxidative phosphorylation balance and normalizes metabolic profiles in fibrotic AEC2, suggesting therapeutic potential (Newton et al., 2021). This has spurred interest in repurposing LDHA-targeted small-molecule inhibitors (currently under investigation in cancer and neurodegenerative diseases (Acharya et al., 2019; Abedini et al., 2011; Gilbert et al., 2015)) for IPF treatment. However, systemic LDHA inhibition carries significant risks: as a central glycolytic enzyme, its broad suppression may disrupt lactate-dependent physiological functions in high-demand organs (e.g., heart and skeletal muscle) and trigger compensatory mechanisms such as ROS-JNK/p38 MAPK pathway activation, potentially worsening fibrosis. These risks mirror challenges observed with other metabolic modulators—for instance, JAK inhibitors (e.g., Ruxolitinib) ameliorate fibrosis but risk immunosuppression (Song et al., 2022), while epigenetic agents like Rhein alleviate renal fibrosis yet may perturb DNA methylation (Middleton et al., 2018; Zhang et al., 2016). Collectively, these findings highlight the need for tissue-specific delivery strategies and comprehensive risk-benefit assessments when targeting LDHA in IPF (Newton et al., 2021; Song et al., 2022; Middleton et al., 2018; Zhang et al., 2016).

#### 3.2.2 Mitochondrial restoration via the PINK1/ATF3/IL-17A axis enhances oxidative glucose utilization

Mitochondrial health is essential for AEC2s to fully oxidize glycolytic pyruvate and meet high ATP demands during repair. In IPF, suppressed PINK1 expression—due in part to ATF3 overexpression—leads to accumulation of damaged mitochondria, a shift toward anaerobic glycolysis, and lactate buildup that fuels fibroblast activation and matrix deposition (Xiao et al., 2022; Moimas et al., 2019; Lawrence and Nho, 2018; Judge et al., 2018). IL-17A further disrupts mitophagy and exacerbates ER stress, reinforcing the glycolytic bias of AEC2s and reducing their regenerative capacity (Bueno et al., 2015; Hartman et al., 2004; Bueno et al., 2018b). Restoring PINK1 via ATF3 knockdown or IL-17A neutralization re-establishes mitophagy, increases mitochondrial respiration, and shifts carbon flux back toward oxidative phosphorylation. This metabolic rebalancing lowers lactate levels, diminishes TGF-β1 activation, and improves AEC2 survival and barrier function, demonstrating that targeting the PINK1/ATF3/IL-17A axis is a viable approach to correct carbohydrate metabolism in fibrotic lungs (Wang et al., 2017; Mi et al., 2011).

#### 3.2.3 Glycolytic and redox interventions to rebalance AEC2 carbon metabolism

Beyond mitochondrial rescue, direct modulation of glycolysis and NAD^+^/NADH balance can further restore AEC2 energy homeostasis. Inhibition of LDHA reduces lactate production, preventing extracellular acidification that drives profibrotic signaling, while supplementation with NAD^+^ precursors (e.g., nicotinamide riboside) enhances sirtuin-mediated mitochondrial biogenesis and promotes pyruvate entry into the TCA cycle (Cui et al., 2022). Targeting glucose uptake via conditional GLUT1 deletion in AEC2s has shown that fine-tuning glucose influx can prevent excessive glycolytic flux and AEC2 apoptosis under stress (Bueno et al., 2015). Moreover, ER chaperones that relieve UPR-induced translational arrest (e.g., 4-phenylbutyrate) synergize with glycolytic inhibitors to reduce ATF4/CHOP-mediated cell death and preserve ATP production through balanced glycolysis–OXPHOS coupling (Yan et al., 2023; Larson-Casey et al., 2020; Kim et al., 2016). (Table 1)

### 3.3 AEC2-based therapeutic strategies for amino acid metabolism

**FIGURE 6:**
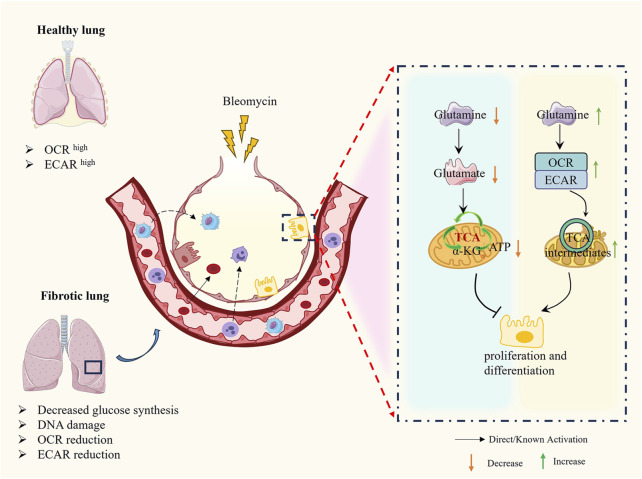
The molecular mechanism of AEC2 glutamine metabolism in PF. Decrease in glutamine and glutamate in damaged AEC2 leads to decrease in α-KG and AKT hindering cell proliferation and differentiation, on the contrary increase in exogenous glutamine restores normal TCA cycling in mitochondria and promotes cell proliferation and differentiation.

Recent studies have shown that the regulation of amino acid metabolism in AEC2 cells plays a key role in the process of pulmonary fibrosis, especially in glutamine metabolism. Glutamine not only supports cellular amino acid synthesis and antioxidant defense as a major nitrogen source but also participates in DNA repair and gene transcription regulation through conversion to α-KG (Pullamsetti et al., 2011; Masri, 2010). By optimizing amino acid metabolism, especially glutamine metabolism, in AEC2 cells, their antioxidant capacity can be enhanced, reactive oxygen species-induced damage can be reduced, and cell repair and regeneration can be promoted. Currently, several studies have been conducted to target glutamine metabolism to intervene in the strategy of treating PF, such as supplementation of glutamine or modulation of related enzyme activities, which can restore the metabolic function of AEC2 cells, reduce cell death, and promote the synthesis of surface-active proteins and the repair of alveolar structures after injuries such as bleomycin (Masri, 2010). In addition to this, the role of glutamine in regulating the TGF-β signaling pathway and lactate metabolism makes it a potential therapeutic target, which may help to alleviate the progression of pulmonary fibrosis (Shaghaghi et al., 2021; Masri, 2010) (Figure 6).

DDAH activity was increased in lung AEC2 cells from mice with bleomycin-induced injury and IPF patients, and inhibition of DDAH could inhibit cell proliferation and induce apoptosis by suppressing cell proliferation in an ADMA-dependent manner, as well as decreasing collagen production in fibroblasts (Gaugg et al., 2019; de la et al., 2020). Therefore, the therapeutic strategy based on amino acid metabolism in AEC2 cells not only provides a new idea for the intervention of pulmonary fibrosis but also lays the foundation for the development of more effective therapeutic approaches in the clinic (Table 1).

## 4 Challenges and the future

Although therapeutic strategies targeting metabolic abnormalities have made some progress in the treatment of pulmonary fibrosis, there are still many challenges and unknowns. Current metabolomic studies in PF predominantly focus on metabolic alterations in myofibroblasts, while research targeting AEC2 requires further in-depth exploration. AEC2 cells serves as the key to maintaining lung physiological function and responding to injury. Metabolic Abnormalities in AEC2, especially in lipid metabolism, mitochondrial function, and lactate metabolism, significantly contribute to cellular injury and the progression of pulmonary fibrosis. However, most existing studies concentrate on individual metabolic pathways, lacking comprehensive investigations into the overall metabolic network and its interactions.

Secondly, current metabolic modulators and targeted therapeutic regimens have not undergone extensive clinical validation, with limited data available on their long-term safety and efficacy. In addition, Given that pulmonary fibrosis is a complex, multifactorial disease, a single metabolic targeting strategy may be insufficient to address its therapeutic challenges fully. Future research should emphasize the cross-regulation of metabolic pathways and their combined effects on the disease course. For example, future studies must elucidate whether and how specific changes in lipid species alter the function of AEC2, and the fluxes of lipid metabolism controlled by AEC2 and its ecological niche supporting the dynamics of lipid biosynthesis, storage, transport, and depletion (via fatty acid oxidation) in the cell remain unclear (Bueno et al., 2018b; Kamp, 2018; Sunaga et al., 2013; Romero et al., 2018b; Proctor et al., 2006). Although lipid supplementation “rescue” studies are expected to open up new therapeutic strategies, it is important to decipher the mechanistic basis for a more robust regenerative response due to membrane biosynthesis (essential for proliferating cells), cellular bioenergetics (diminished with aging), or epigenetic programs (regulated by lipid signaling intermediates) that are lipid-supported. In addition, it is hypothesized that ER stress and metabolic abnormalities combine to cause damage to AEC cells and enhance their susceptibility to environmental damage (Wang et al., 2017; Barreiro et al., 2019; Feng et al., 2024; Yan, 2019). Although AEC2 appears to be the primary target of ER stress in lung fibrosis, other cells, including fibroblasts, can regulate the fibrotic process through UPR activation. Clarifying which UPR pathways are most critical and how ER stress-induced cellular phenotypes (inflammation, apoptosis, or EMT) regulate fibrotic remodeling is essential for designing effective therapies to limit the effects of ER stress on pulmonary fibrosis.

It is worth noting that acute lung injury (ALI) and pulmonary fibrosis PF share significant similarities in metabolic abnormalities: both are characterized by mitochondrial damage, ROS accumulation, and metabolic reprogramming. For example, the BCAP31/PINK1/Parkin pathway alleviates inflammation and oxidative stress by restoring mitochondrial autophagy in ALI (Vij, 2020; Jiang et al., 2024; Du et al., 2024), whereas downregulation of PINK1 in PF leads to impaired mitochondrial clearance and exacerbates the fibrotic process (Zhu et al., 2024). This commonality suggests that metabolic intervention strategies targeting mitochondrial autophagy have broad spectrum applicability. Future studies are needed to further validate the efficacy of such strategies in different lung disease models and to explore tissue-specific delivery systems, such as lipid nanoparticles targeting AEC2, to reduce systemic toxicity.

**FIGURE 7:**
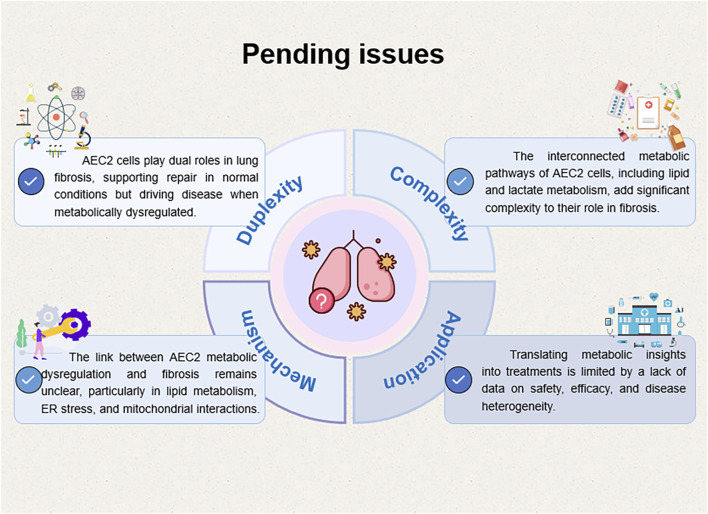
A diagram of several key scientific issues that remain to be addressed in the future.

In summary, while significant advancements have been made in understanding the role of metabolic dysregulation in pulmonary fibrosis, comprehensive studies addressing the intricate metabolic networks and their interactions within AEC2 cells are necessary. Additionally, rigorous clinical validation of metabolic modulators and combination therapies targeting multiple metabolic pathways may offer more effective treatment options for pulmonary fibrosis (Figure 7).

## 5 Conclusion

Despite significant advances in our understanding of the pathobiology of PF over the past 2 decades, existing therapeutic strategies are primarily aimed at slowing down the disease progression without achieving a cure. Therefore, there is an urgent need to develop better and safer alternatives.

Abnormalities in the metabolism of AEC2 are closely associated with the development of PF, particularly disruptions in lipid metabolism, mitochondrial function, and lactate metabolism. Targeting these metabolic pathways holds great potential for the treatment of PF. For example, Liang et al. (2024) proposed that abnormal lipid metabolism leads to AEC2 dysfunction, a hallmark of PF. Notably, their work demonstrated that the regenerative capacity of aged AEC2 cells in a 3D organoid model could be rejuvenated through lipid supplementation or activation of PPARγ agonists. Similarly, Sunaga et al. (2013) showed that Elovl6 levels were reduced in PF lung tissues, and Elovl6 deficiency in mice resulted in spontaneous thickening of the alveolar wall and increased susceptibility to bleomycin-induced pulmonary fibrosis. Romero et al. (2018b) reported that SCD1 levels are reduced in PF lung tissues and that pharmacological inhibition of this enzyme leads to ER stress and induces pulmonary fibrosis remodeling in mice. Although the above studies did not specifically elucidate the corresponding mechanisms of lipid metabolism in AEC2, in conjunction with studies in other areas, we believe they may have direct clinical implications. For instance, in diabetes, alterations in lipid metabolism favor the synthesis and accumulation of triglycerides and cholesterol, which are associated with elevated transforming growth factor β levels and the development of tubulointerstitial fibrosis (Proctor et al., 2006). Furthermore, in the field of obesity, it has long been recognized that organ dysfunction is at least partially due to the accumulation of saturated fatty acids outside of adipose tissue, particularly in the cell membranes of cardiovascular tissue (Barreiro et al., 2019).

Moreover, lactate metabolism and mitochondrial energy metabolism targeting AEC2 have gradually become emerging research hotspots. Lactate metabolism may play a key role in fibrosis by regulating TGF-β signaling and mesenchymal cell function (Feng et al., 2024). Mitochondrial dysfunction leads to disturbed energy metabolism and increased oxidative stress, further contributing to fibrosis progression (Yan, 2019; Vij, 2020; Jiang et al., 2024; Du et al., 2024). Current studies have found that restoring mitochondrial function and improving lactate metabolism can effectively slow the progression of pulmonary fibrosis. Additionally, targeting autophagy, antioxidants, or metabolite supplementation (e.g., glutamine and metformin) has shown promise in mitigating fibrosis (Zhu et al., 2024; Cheng et al., 2021). Furthermore, emerging approaches such as stem cell-based therapies (e.g., microfluid-ic-templated stem cell microcapsules) have shown potential to reverse fibrosis in animal models, although their safety and long-term efficacy require further validation (Wu et al., 2022).

In conclusion, ongoing research continues to demonstrate that metabolic dysregulation is a key factor in the pathogenesis of pulmonary fibrosis. Therefore, drugs targeting various aspects of cellular metabolism—including glycolysis, mitochondrial oxygen consumption, and lipid metabolism—should be actively pursued as potential treatments for this debilitating disease.

## Abbreviations

PF, Pulmonary fibrosis; AEC, Alveolar Epithelial Cell; IPF, Idiopathic pulmonary fibrosis; ILD, Interstitial Lung Disease; FDA, Food and Drug Administration; AEC1, Alveolar Epithelial Cell Type I; AEC2, Alveolar Epithelial Cell Type Ⅱ; ER, Endoplasmic reticulum; MFN1, Mitofusin 1; MFN2, Mitofusin 2; LDH, Lactate Dehydrogenase; ACSS3, Acyl-CoA Synthetase Short Chain Family Member 3; APOA1, Apolipoprotein A-I; PA, Palmitic Acid.
